# A Low Cost/Low Power Open Source Sensor System for Automated Tuberculosis Drug Susceptibility Testing

**DOI:** 10.3390/s16060942

**Published:** 2016-06-22

**Authors:** Kyukwang Kim, Hyeong Keun Kim, Hwijoon Lim, Hyun Myung

**Affiliations:** 1 Urban Robotics Laboratory, Korea Advanced Institute of Science and Technology, 291 Daehak-ro, Daejeon 34141, Korea; kkim0214@kaist.ac.kr; 2 Department of Mechanical Engineering, Korea Advanced Institute of Science and Technology, 291 Daehak-ro, Daejeon 34141, Korea; hkkim1227@kaist.ac.kr; 3 School of Electrical Engineering, Korea Advanced Institute of Science and Technology, 291 Daehak-ro, Daejeon 34141, Korea; wjuni@kaist.ac.kr

**Keywords:** mycobacteria, nitrate reductase assay, drug susceptibility test, sensor node, Bluetooth low energy beacon, 3D-printer, low power, low cost

## Abstract

In this research an open source, low power sensor node was developed to check the growth of mycobacteria in a culture bottle with a nitrate reductase assay method for a drug susceptibility test. The sensor system reports the temperature and color sensor output frequency change of the culture bottle when the device is triggered. After the culture process is finished, a nitrite ion detecting solution based on a commercial nitrite ion detection kit is injected into the culture bottle by a syringe pump to check bacterial growth by the formation of a pigment by the reaction between the solution and the color sensor. Sensor status and NRA results are broadcasted via a Bluetooth low energy beacon. An Android application was developed to collect the broadcasted data, classify the status of cultured samples from multiple devices, and visualize the data for the end users, circumventing the need to examine each culture bottle manually during a long culture period. The authors expect that usage of the developed sensor will decrease the cost and required labor for handling large amounts of patient samples in local health centers in developing countries. All 3D-printerable hardware parts, a circuit diagram, and software are available online.

## 1. Introduction

*Mycobacterium tuberculosis*, a rod-shaped bacterium with an acid-fast property, is a well-known cause of tuberculosis, a disease generally transmitted by aerial infection and notorious for its highly contagious properties and associated mortality [1]. The World Health Organization (WHO) estimates that including patients and carriers about one-third of the world population is infected [2]. About 9.6 million new outbreaks occur annually, leading to about 1.5 million annual deaths [3]. Recently, the appearance of multi-drug resistance bacteria due to the abuse of antibiotics and the observation of fatal complications in AIDS patients have made early diagnosis of tuberculosis more important [4].

Previous studies focused on the detection of mycobacteria. Many methods including ZN-stained sputum smear microscopic imaging [5], culture [6], molecular diagnosis methods such as polymerase chain reaction (PCR) [7], and immunological methods such as enzyme-linked immunosorbant assay (ELISA) [8] have been developed. The diagnosis methods focus on antibiotic drugs for tuberculosis treatment, and the diagnosis can be directly linked to a prescription and treatment. However, an increase of multi-drug resistance of mycobacteria has made treatment of tuberculosis patient with only diagnosis information difficult, thus making drug susceptibility tests (DST) essential. However, a drug susceptibility test has to assure that mycobacteria do not grow when a certain drug was given; for this purpose bacteria have to be cultured, even though they have a very slow growth rate (about 16 h doubling time).

Many culture techniques have been developed to decrease the time, cost, and labor of drug susceptibility tests. Automated commercial mycobacteria growth checking systems such as BACTEC [9] that use liquid media to promote mycobacteria growth were developed. However, liquid media are more vulnerable to contamination and this system is too expensive for developing countries where the largest portion of tuberculosis patients exists. Early growth detection methods using a microscope such as Microscopic Observation of Drug Susceptibility (MODS) [10] or Thin Layer Agar (TLA) [11] have also been developed. These methods have decreased the time and cost gradually, but a microscope still costs more than 5000 US dollars [12]. Even if microscopes are available, they are not resilient devices that can be operated at remote areas with limited resources or infrastructure. Furthermore, the user has to be trained to distinguish microscopic images of the growing *M. tuberculosis* from other bacteria or fungi including other *Mycobacterium* species or fungi. This also requires additional time. A MODS image training website [13] exists, but access to these data is limited to the areas where access to the internet is possible. 

**Figure 1:**
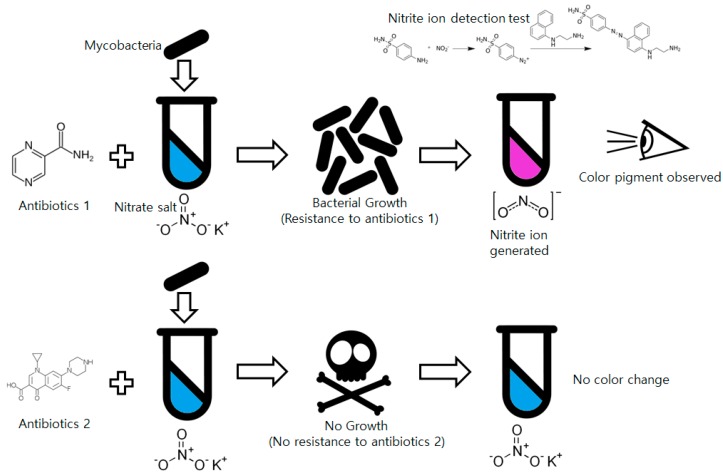
Process of the drug susceptibility test of mycobacteria using the nitrate reductase assay.

Among low-cost, non-commercial mycobacteria drug susceptibility testing methods, the nitrate reductase assay (NRA) is promising from the perspective of simplicity [14]. Almost every *Mycobacterium* species has an enzyme that can reduce nitrate ions (NO_3_^−^) into nitrite ions (NO_2_^−^). Mycobacteria cultured with nitrate salt-including media generate nitrite ions in the culture media that can be detected by simple chemical tests such as a Griess test. The Griess reagent using *N*-(1-naphthyl)ethylenediamine dissolved in acetic acid and sulfanilic acid is used for nitrite ion detection [15]. The two solutions react with the nitrite ions and form a visibly distinguishable red-purple colored azo dye. Compared to other methods such as MODS or TLA, NRA is much simpler for non-trained technicians to use by simplifying the whole drug susceptibility test into a presence/absence test (P/A-test) for nitrite. Because of these advantages, even though NRA is not suitable for tuberculosis diagnosis, it is widely used for drug susceptibility tests and approved by the World Health Organization [16]. The overall process of the NRA is shown in Figure 1.

Although NRA is useful for low cost DST of the mycobacteria for non-skilled technicians, it is a labor intensive job as it requires handling many (number of patients × number of available antibiotics) samples. Even a simple culture-detection process of the fast-growing bacteria such as *E. coli* can be labor intensive [17] and mycobacteria requires more time to grow than fast growing bacteria (at least 14 days are required for the NRA test [14]), making handling a large amount of culture samples into a very hard and labor intensive job. Also, manual examination of bacteria growth becomes more cumbersome in the actual clinical field: as mycobacteria generally cause massive infections in local communities, having the health authority test all candidates’ samples would impose a huge burden.

In this study, a low-cost sensor system was developed that automatically performs NRA to check the drug resistance of mycobacteria. A culture bottle containing Ogawa media with a nitrate salt is combined with a linear motor that controls a syringe to inject the nitrate-detecting chemicals into the culture bottle after a certain incubation time has passed. The appropriate time for the NRA test operations is measured by a microcontroller. The color sensor detects the result of the NRA and sends it to the end user via a Bluetooth low energy (BLE) beacon. Other critical information such as temperature data is also included in the transmitted packet. The sensor node was designed to consume a small amount of battery power and recharge from a solar panel for usage in environments with limited resources such as local areas of developing countries.

## 2. Materials and Methods

### 2.1. Bacterial Strains and Culture

The target bacterium in this research is *Mycobacterium tuberculosis*; however, the high pathogenicity and slow growth rate of this bacterium hinder culture, sample preparation, and other required processes. The purpose of our system was to develop the sensor which can automatically sense red color pigment formation rather than reproducing a NRA result with the *M. tuberculosis*. *Mycobacterium smegmatis*, a non-pathogenic mycobacteria model species that has a relatively fast doubling time and high genetic/biochemical similarity (such as an ability to reduce nitrate) with *Mycobacterium tuberculosis,* was used as an alternative due to the safety issue. *M. smegmatis* can also generate a red pigment during the nitrate reductase assay if cultured with the nitrate ion, mimicking the role of the *M. tuberculosis* during the DST with NRA. *M.* *smegmatis* MC2 155 strain [1] was kindly provided by the Department of Microbiology, Medical School of Chungnam National University in South Korea. The obtained strain was cultured with 2% Ogawa medium in a 36 °C incubator [2].

### 2.2. Nitrate Reductase Assay Protocol

A number of commercially available nitrite ion detection kits were examined, and an API Nitrite Test Kit (Mars Inc., McLean, VA, USA) [18], a single-solution kit that has a 0 to 5 ppm detection range, was finally selected. For the nitrate reductase assay, 100 μL of 0.1 M (about 10 mg/mL) potassium nitrate (Junsei Chemical Co., Ltd., Tokyo, Japan) [19] solution was spread on the Ogawa media. After the solution was absorbed by the media, bacterial strains were streaked on the media. For *M.* *smegmatis*, 3 days of incubation were required. After incubation, a detection solution consisting of 100 μL of the API nitrite test kit mixed with 5 mL of distilled water (DW) was prepared and injected (1.5 mL to 4 mL). Colored pigment formation of the injected detection solution due to concentrated nitrite ions in the media was checked with a sensor.

### 2.3. Development of the Sensor Node Device

#### 2.3.1. Hardware Configuration and Construction

**Figure 2:**
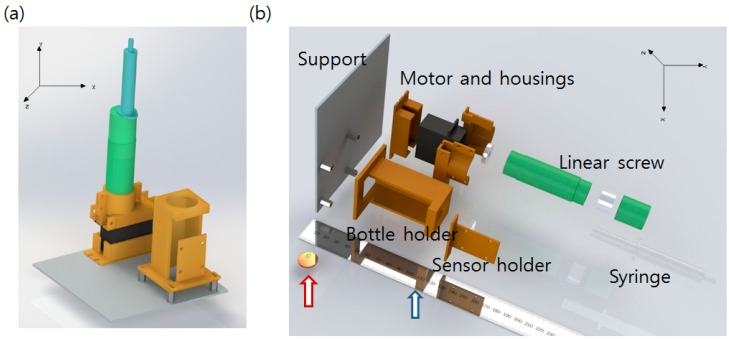
(**a**) Designed hardware component. Orange colored parts are 3D-printed. Empty space at the left side is reserved for the circuit; (**b**) Exploded view of the hardware system describing how the components are assembled. A centimeter scale ruler (Blue arrow) and one cent (Red arrow) coin are added in the image for scale comparison.

The hardware consists of two parts: an Ogawa bottle holder that assures the color sensor remains at an appropriate distance from the bottle and a linear DC motor that injects the detection solution with a syringe after the incubation is finished. A commercial syringe pump is very expensive, and it should be optimized for injection of very small amounts of liquid. The system developed in this study requires fast injection of a relatively large amount of liquid (mL scale). We therefore developed a low-cost linear DC motor combined with a syringe. A glue stick with a screw function was purchased, and the glue was removed. The DC motor was attached at the rear, screw handle part of the glue stick and the syringe was fixed at the stopper part of the glue stick. When the DC motor operates and turns the screw handle, the driving part of the glue stick originally used to supply glue moves forward. The piston of the syringe is pushed by the driving part, and liquid inside of the syringe is ejected. After ejection, the syringe can be uncorked with the stopper and re-filled again. Reverse turn of the DC motor can rewind the driving part to the original position, making the system reusable. Self-designed, 3D-printed additional parts were fabricated to fix the link between the glue stick and the DC motor. The top of the Ogawa media bottle was drilled and a rubber cork to allow piercing by the syringe needle was applied. The syringe and the needle were linked with a silicon tube. All hardware parts including the Ogawa/color sensor holder were self-designed with SolidWorks 3D CAD software [20] and fabricated using a 3D printer (a MakerBot2 printer with an ABS filament, resolution of 0.2~0.3 mm with 50% infill [21]). The overall hardware component design and an exploded view image are shown in Figure 2.

#### 2.3.2. Circuit Design and Programming

For rapid prototyping and easy access, Arduino [22] was used as the main microcontroller of the developed sensor system. To minimize power consumption, an Atmega328 Atmega328 (Atmel, San Jose, CA, USA) containing an Arduino bootloader with the least required circuit elements was used as the mainboard. The sensor node was programmed with the Arduino IDE. Chip-less Arduino Uno board’s Reset and Universal Asynchronous Receive and Transmission (UART) ports were connected to the Atmega328 chip of the mainboard when it was programmed.

A temperature sensor (LM35DZ, Texas Instruments, Dallas, TX, USA) and a programmable color light-to-frequency converter sensor module (TCS230, AMS-TAOS USA Inc., Plano, TX, USA) [23] were added. This color sensor consumes about 50 mA. To reduce the power usage, the color sensor was turned off when it was not used. The power line of the color sensor was not connected to the regulated 5 V power source; instead it was connected to a motor driver (BA6208, ROHM semiconductor, Kyoto, Japan) that supplies power to the sensor when an operation signal comes from the microprocessor. The second channel of the motor driver was connected directly to the DC motor pump.

Collected sensor data are broadcasted via a Bluetooth low energy (BLE) beacon. A BLE chip (BoT-CLE 110, Chipsen, Gyeonggi-do, Korea) was placed and powered with a 3.3 V coin cell battery. For data broadcasting, the beacon has to be powered continuously, but a 3.3 V regulator consumes over 10 mA to power the beacon. An additional battery for the beacon was therefore installed and a 3.3 V regulator (LM3940, Texas Instruments, Dallas, TX, USA) and a logic level converter (TSB12009, Sparkfun electronics, Niwot, CO, USA) powered when sensor data are updated were placed to link the communication line between the microprocessor (5 V logic) and the BLE chip (3.3 V logic).

**Figure 3:**
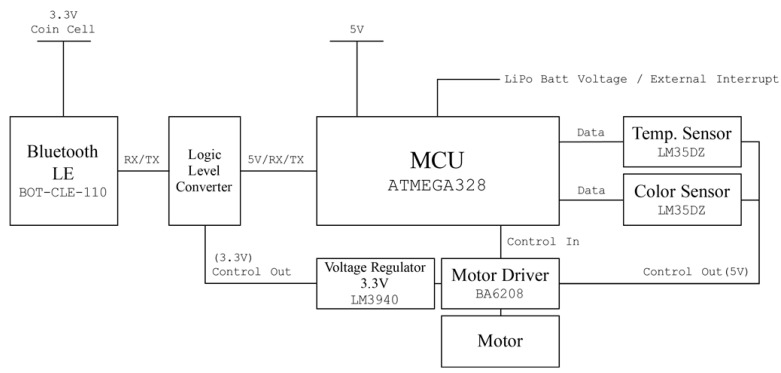
Circuit diagram of the designed sensor system.

All circuits were built in a single board (the circuit diagram is shown in Figure 3) and stacked over the Arduino compatible solar cell shield (Solar Charger Shield v2.0b from Seeed Technology Limited, Shenzhen, China) [24]. This shield provides 5 V regulated output from a 3.7 V, 1 cell lithium polymer (Li-Po) battery and charges it when enough light is irradiated to the attached solar cell, which can increase the operation time of the sensor node in a harsh situation. All circuit elements and a wiring diagram are shown in Figure 3.

### 2.4. Development of the Periodic Polling Device

**Figure 4:**
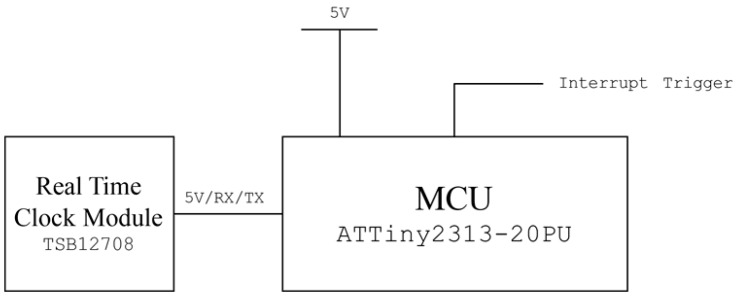
Circuit diagram of the designed off-line periodic polling device.

A periodic polling device that triggers the sensor node for every user-defined time was developed using a Real Time Clock (RTC) module (TSB12708, Sparkfun electronics, Niwot, CO, USA) and Attiny2313 microprocessor chip (Atmel). The RTC module’s square wave output pin was connected to the external interrupt pin of the Attiny2313 chip to trigger the chip’s pulse counter at every 1 s. A square wave pin was also connected to the voltage line as it is an open drain port. When a pulse count number meets the pre-programmed threshold, the polling device triggers the 5 V output to trigger the external interrupt of the sensor node and resets the time counter to prevent overflow. A circuit diagram of the polling device is shown in Figure 4.

If the user can access an on-line network and stable electrical infrastructure, more accurate time measurement is possible. For accurate time measurement, another polling device was developed with Arduino and an Arduino compatible Wi-Fi shield (Arduino, Ivrea, Italy), which enables the microcontroller to access the wireless network. Via a wireless network, the microcontroller can access the network time server by Network Time Protocol (NTP). Time elapse in seconds can be measured accurately by receiving a standard time server’s data. If the user-defined time exceeds, the on-line polling device generates a trigger signal like the off-line RTC-based polling device.

## 3. Results and Discussion

### 3.1. Nitrate Reductase Assay

Commercial Griess test kits for laboratory usage are available; however, these reagent kits are rather expensive (about 200 US dollars) and access is limited as chemical compounds are toxic. Also, automatically performing a Griess test is complicated as most kits consist of two reagents, which requires more liquid handling devices. Instead, we used an API nitrite detection kit available commercially for nitrite ion detection in food or aquariums. These kits are cheaper (about 12 US dollars), easy to store (storage at room temperature is possible), and less toxic.

**Figure 5:**
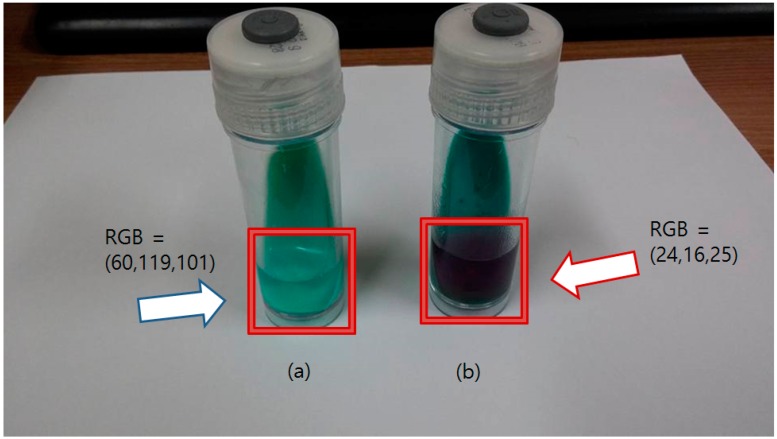
Result of the Nitrate Reductase Assay using API Nitrite Kit based detecting solution. (**a**) The bottle at the left side is the blank sample (nothing cultured), which shows the detecting solution remains clear and unchanged (blue arrow); (**b**) The bottle with the positive control is at the right side, showing a purple solution (red arrow). The RGB values indicate the average RGB values of the liquid region.

The nitrite ion detecting solution was injected into the Ogawa media bottle where the mycobacteria were cultured with potassium nitrate. Fresh Ogawa media (nothing cultured) and mycobacteria cultured without potassium nitrate were used as the negative control and blank, respectively. As soon as the detecting solution was injected, the color of the solution changed from clear sky-blue to a deep purple color. In the blank and negative control, the color remained unchanged. The result of the NRA with the API Nitrite Kit-based solution is shown in Figure 5.

High concentrations of the nitrite ion were detected when mycobacteria were co-cultured with the potassium nitrate solution. The injected detecting solution rapidly changed to dark purple. The changed color was sustained for about 5 min and slowly became dark brown from the bottom region of the bottle. The sustaining time of the pigment does not affect the performance of the sensor, as 5 min was enough time for the color sensor to check the solution color.

An unexpected color pigment formation of the nitrite ion detecting kit was observed during the experiment. Many nitrite ion detection kits rely on the formation of the red colored azo dye; however, when nitrite ions exceed a certain concentration, the red pigments are removed and yellowish colored products are generated, changing the solution color to pale green (API Nitrate Kit) or vivid yellow (other Griess test based kits). The exact mechanisms are not known, but an excessive amount of potassium nitrate (causing too high a concentration of the nitrite ion) can cause an error during the NRA test.

### 3.2. Assembly and Operation of the Sensor System

**Figure 6:**
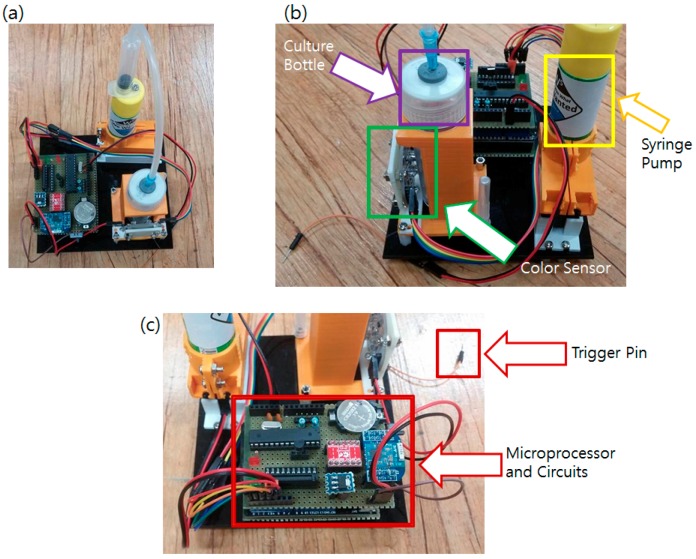
Developed main sensor node. (**a**) Built sensor system; (**b**) Closer view of the color sensor viewing the culture bottle; (**c**) Closer view of the electronic parts.

All circuits and hardware components of the main sensor node were assembled together and firmware was programmed. The complete main sensor system is shown in Figure 6.

**Figure 7:**
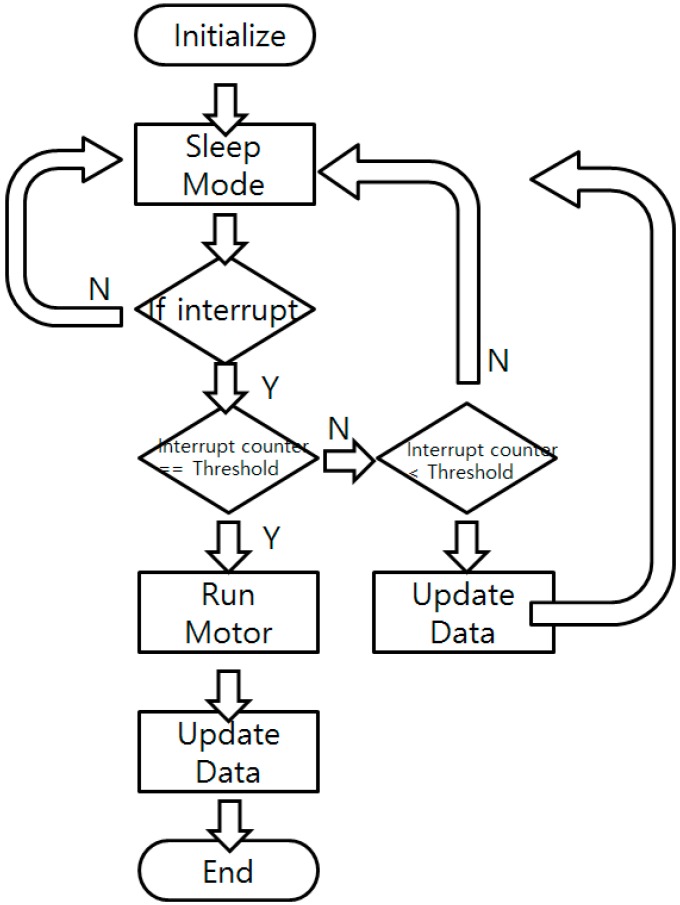
Software flow chart of the main sensor node.

To reduce the power consumption, the sensor node is programmed to stay in sleep mode (power down mode), only receiving external interrupts. When external interrupts are given, the system wakes up and disables all the interrupts to prevent possible confusion. It increases the interrupt counter and reports the sensor status such as battery voltage, temperature, and color sensor reading value to check whether the culture process or the system is in a normal state. Updated data are sent to the BLE beacon to change the broadcasted data. If the interrupt counter’s value equals the pre-defined user-set threshold, the motor operates and the nitrite ion detection solution is injected into the culture bottle to perform the NRA test. The color of the bottle is checked and the broadcasted value is fixed after the motor operation. When all the tasks are done, the sensor node turns down every power line, enables the interrupt, and goes back to the sleep mode. A flow chart of the software is shown in Figure 7.

A positive control sample (mycobacteria cultured with potassium nitrate) and a blank sample (nothing cultured) were introduced to the sensor node and their functionality was examined. The color sensor (TCS230) attached at the sensor node is equipped with three color filters (Red, Green, and Blue) and designed to give a pulsed signal output based on the color frequency that it receives. If the red filter is selected, the frequency of the output pulse decreases as more red lights are radiated. Change of the pulse is measured by the microcontroller attached to the sensor node. During operation, all interrupts are disabled when the sensor node is awakened from the sleep mode to prevent confusion between interrupt vectors. Instead, the General Purpose Input and Output (GPIO) pin is connected to the sensor output and the change of the High/Low signal is counted. The time to reach at certain counts (number of pulse counts) was measured (milliseconds passed to receive 1024 pulse change, for example). Event repeats per second were calculated from the data and the frequency was measured. When the positive control was placed, the sensor node could receive about 5 kHz to 8 kHz output while the blank sample gave about 11 to 15 kHz. The repeated test showed negative samples gave frequency output near 15 kHz, and at least 11 kHz, while the positive samples always gave values lower than 9 kHz. The API Nitrite detection Kit showed red pigment generation when nitrite ion concentration exceeded 0.5 ppm. The sensor output started to drop to 10 kHz range. The positive result of the NRA generally shows a lot higher concentration of nitrite, showing that the sensor can successfully read nitrite changes in the culture bottle.

Compared to a camera, another candidate for a color pigment formation monitoring sensor, the proposed color sensor is much cheaper and can be operated with less battery power or infrastructure. In case of commercial UVC webcams, an Operating System (OS) equipped with video codec and a USB driver are required. An embedded Linux board can fulfill these requirements, but it consumes a lot more energy than the 8 bit microcontroller used in this research. Also, power saving mode of the OS equipped system is much more complicated to operate compared to the microcontroller. To culture the bacteria, the sensor node should be placed in the incubator or insulated boxes, which has no light source for the webcam. The color sensor has a white LED attached as a default, which emits a proper amount of light for color detection without any external devices for light. Overall, for a simple color detection job, the color sensor with basic functions was more appropriate than a camera-based sensor.

### 3.3. On-line and Off-line Periodic Polling Device

The main sensor node described above does not have internal clocks and instead relies on a periodic external interrupt signal to operate. The developed sensor node is compatible with any timing device if the device can periodically trigger the external interrupt pin. Generally, a RTC or a network synchronized timer is used. However, a network device requires data connection infrastructure and consumes relatively large current to operate. A RTC can operate for about a year with a single coin cell battery, but many low priced RTCs available on the market show low stability such as unexpected reset of time settings. Instead of receiving data from the RTC, we used a pulse counter to make the timing device. Many RTC modules have a square wave output (often denoted as SQW) pin that generates a constant 1Hz square wave. By counting the pulse input, the microprocessor can continuously receive the exact timing signal without relying on unstable memory data of the RTC module.

**Figure 8:**
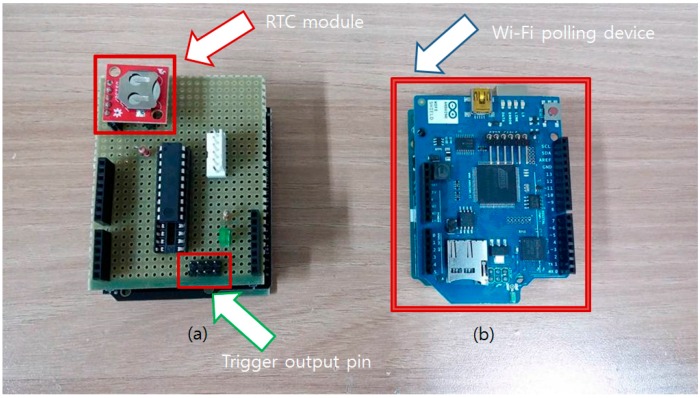
Developed on-line/off-line polling devices. (**a**) The off-line RTC-based polling device is shown at the left side. Red arrow indicates that the RTC generates 1Hz wave for the time counting. Pinouts pointed by the green arrow are trigger output pins when the timer reaches the given time; (**b**) The blue board at the right side is a network time-based on-line polling device. Blue arrow shows a Wi-Fi shield that enables access to the time server.

To minimize power consumption, the Attiny2313 chip goes into the sleep mode (power down mode) when not triggered by the square wave from the RTC module. Also, an internal clock of 128 kHz instead of external crystal was used to drop the clock speed as much as possible. On-line polling devices are not equipped with power saving functions as it is assumed that the sensor is used in a resource-rich environment. Two completed polling devices are shown in Figure 8.

### 3.4. Power Consumption Measurements

Power consumption of each element of the sensor system was measured using a current sensor (TSS12040, Sparkfun electronics, Niwot, CO, USA). Each component of the circuit was isolated and consumption of the current was measured when it was in sleep mode or active. The results are shown in Table 1.

When the main sensor node is in sleep mode, only the microcontroller in the power down mode, temperature sensor (LM35DZ), motor driver (BA6208), and BLE beacon with an external coin battery are powered. LM35DZ and BA6208 consume excessively small current to be measured with the current sensor. The BLE beacon consumes about 300 μA but it increases to about 1500 μA when the transmission power level increases (the lowest transmission level was used). About 500 μA is consumed from the main battery and about 300 μA is drained from the 3.3 V coin cell battery for the BLE when the sensor node is in the sleep mode. When the sensor node is activated, about 0.1 A for checkup process and 0.8 A for motor operation (used only once during DST) is consumed. Sleep mode and the deactivation circuit using the motor driver to power elements gradually decrease the power consumption compared to the fully active mode. The solar charger shield’s 3.7 V to 5 V up regulation circuit consumes 2.5 mA constantly. However, the solar charger shield is used when an external solar power source is available; its charging ability cancels out its native current consumption. A single celled 3.7 V lithium polymer (Li-Po) battery supplying about 2500 mAh to 4000 mAh is commercially available. With the current consumption amount of our sensor system, it is expected that the user can run the sensor for a very long time without charging or changing batteries, thereby increasing user convenience.

### 3.5. Sensor Data Collection and Visualization

**Figure 9:**
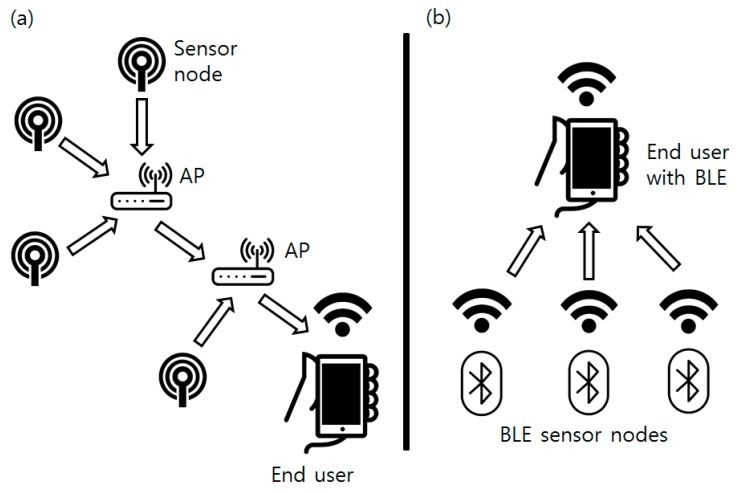
(**a**) Network configuration of the classical mesh topology sensor networks; (**b**) Network configuration used in this research.

Collected sensor data are broadcasted by the BLE beacon attached at the sensor node. Classical sensor networks are generally developed in the form of a multi-hop network: a mesh-shaped network composed of routers and end nodes [25]. This type of network is used for stability of the whole network system and carrying information to the end user located at greater distance than the single node’s coverage. However, the bacterial culture process is usually done in a single room or facility with concentrated sensor devices where the broadcasting distance can be easily covered by a single node. Instead of a classical star or mesh topology network, this research used a simpler form of a broadcast-listen network based on BLE. The BLE beacon consumes a very small amount of energy and can be operated by a coin cell battery if it operates in a “broadcasting mode.” Instead of two-way packet transmission, data are broadcasted one-way from the BLE beacon. Structural differences between the mesh network and the BLE network are shown in Figure 9.

The BLE beacon’s data are composed of two different parts: a beacon ID and advertising data. Each of the main sensor nodes has its own fixed maximum 8-byte length ID (BM-W5xxx where x can be any 8-bit ASCII character) and any ASCII code character was used in this research). The main sensor node changes advertising data (maximum 11 bytes) of the BLE beacon to broadcast an appropriate message to the end user. In this research, “Hz-M-T-B-C” was used as the data protocol, where “Hz” is a two-byte string for color sensor frequency (“11” if the color sensor output is 11 kHz), “M” for 1 byte string for the motor operation flag (0 for not operated yet, 1 for operated), “T” for 1 byte character converted temperature sensor reading (Celsius temperature such as an integer value of 33 is converted into character value by typecasting), “B” for the 1 byte character converted battery voltage, and “C” for 1 byte character converted accumulated interrupt counter increases when the main sensor node is triggered. The receiver, which can read broadcasted BLE signals, decodes the given information and checks the status of each sensor system without a complicated protocol or network setup. Compared to Zigbee, the most common protocol for a classical mesh sensor network, the BLE consumes less energy and is compatible with a smartphone. Also, the initial settings of the network such as the router designation or mesh formation are not required. A non-skilled user can easily set up the BLE network by powering each end node and scanning it with a BLE compatible device such as a smartphone.

**Figure 10:**
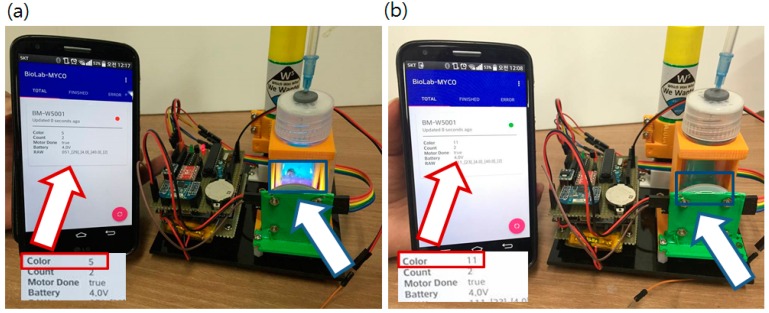
Operating sensor node with the android application. White boxes under the red arrows are closer view of the screen. Red boxed number after the *Color* label shows kHz level frequency value output from the color sensor. (5 indicates 5 kHz output) (**a**) Status reported from the App. (red arrow) when a positive sample (purple liquid in the blue box indicated by blue arrow) was introduced. Dropped color value from 11 kHz to 5 kHz means red pigment generated; (**b**) Report changed when a blank sample (clear-blue liquid in the blue box pointed by blue arrow) was introduced. Color value did not changed and remains still at 11 kHz. Red/Green circled indication mark (red for positive) changes and sensor values are updated.

The authors also developed an Android application (App) to scan nearby sensor nodes and classify the status based on the received sensor data. The Android App decodes and shows each parameter of the received data from the sensor node. It also filters the node status such as a node that finished operation (NRA DST has done) or reporting error data. Some criteria such as low battery voltage or update errors are used to select a node with bad status. Among many culture bottles, the user can monitor and identify problematic nodes without examining each culture bottle directly. Operation of the App with the main sensor node is shown in Figure 10.

**Figure 11:**
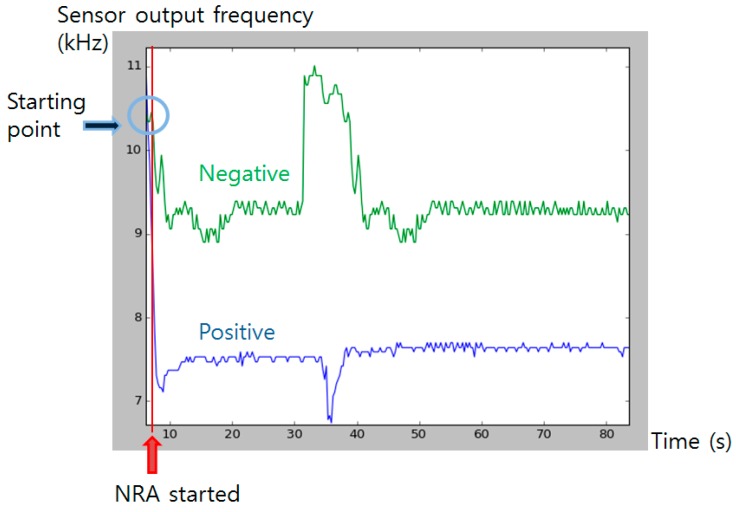
Color sensor output profiling after the injection. Blue graph shows positive signal (color pigment formed) and green line shows negative control. Red line indicates the time that NRA solution was injected. Blue circle indicates the initial sensor output value at the starting time.

The output from the color sensor was monitored for a certain period to obtain profile of color change after the NRA reaction has initiated (Figure 11). After the chemical reaction started by injection of the detecting solution, the frequency output from the color sensor rapidly dropped from about 11 kHz to near 7 kHz. The color pigment formed until 35 s has passed, and slowly started to bleach as time passed. The pigment fully bleached after about two hours (data not shown). With the used color sensor, whole range and magnitude of color pigment formation difference was detectable.

## 4. Conclusions

In this research, a sensor system was developed that periodically monitors the state of a mycobacteria culture bottle and operates a nitrate reductase assay after the culture process finishes to check for drug susceptibility. An Arduino-based low power operation circuit (which consumes a μA level of current in the sleep mode state) that goes into sleep mode when not in the operation mode with a solar panel charging system was developed for long term operation of the sensor at remote areas with limited resources. The sensor system transmits the culture status and the result to an Android application via a BLE beacon. The Android application automatically classifies and reports samples after the culture process or samples in limited condition. The end user can scan results from the BLE beacon with a smartphone without setting up complicated mesh networks.

For easy and low cost replication, hardware parts were fabricated by using 3D-printers and commercial gadgets. Simple sensors and electrical components were used to lower the price of the sensor system (the prototype costs about 60 US dollars for the core parts, and about 100 US dollars including the solar charging system). Cost comparison is shown at Table 2. All components including 3D printable files and the software codes are open to the public via the open source community (Software and circuit at the Github: [26], Hardware components at the Thingiverse: [27], and Demonstration video at the Youtube: [28]).

As future work, we are considering modifying the User Interface (UI) of the sensor node. It is easy to install and use the sensor, but user-defined parameters including culture time should be re-programmed if the user wants to change them. Current settings are optimized for the NRA DST of *M. tuberculosis*. However, other rapidly growing mycobacteria (RGM) species can also cause many infectious diseases. Allowing easier change of the culture time and the polling period can help users to optimize the sensor system for their own purposes.

Current color sensors cannot detect contamination (fungi or early growth of unexpected bacteria on the culture media). An unexpected drop or increase of the color sensor output frequency was observed, but no specific up/down output patterns were found. If the formation of a white fungi or colony can be detected, the sensor node can alert the error during the culture process specifically.

## Figures and Tables

**Table 1:** Current consumption of each component of the sensor system.

Element	Current Usage	Note
Atmega328	500 μA	Sleep mode
Atmega328	0.02 A	At Active
LM35DZ	N/A	Miniscule
BLE *	300~1500 μA	Tx power level
BA6208	N/A	Miniscule
LLC *	N/A	Miniscule
3.3 V regulator	10 mA	At Active
Color sensor	0.02 A	At Active
Solar Sheild	2.5 mA	
Motor	0.7 A	At Active
Attiny2313	100 μA	

* Indicates 3.3 V input.

**Table 2:** Cost comparison between the proposed method and the MODS.

Automated NRA	Cost (USD)	MODS	Cost (USD)
Smartphone ^1^	200	Inverted Light Microscope ^2^	2500
Reagent ^3^	10	Reagents	280
Ogawa Medium	60	Middlebrook 7H9 Medium ^4^	250
Electronics	100	Manual - Not Applicable	-
Total	370	Total	3030

^1^: Motorola Moto G (2015); ^2^: VanGuard Trinocular Inverted Brightfield Microscope; ^3^: API Nitrate Test Kit; ^4^: Hardy Diagnostics TB MODS TB50 Kit.
